# Propofol-sparing effect of different concentrations of dexmedetomidine: Comparison of gender differences

**DOI:** 10.1007/s00101-018-0506-6

**Published:** 2018-11-07

**Authors:** Ming Xiong, Zhao -Xin Zheng, Zu-Rong Hu, Jing He, Uchenna Madubuko, Dennis Grech, Xing-An Zhang, Bo Xu

**Affiliations:** 1 Department of Anesthesiology, General Hospital of Southern Theatre Command of PLA, 510010 Guangzhou, China; 2 Department of Anesthesiology & Peri-Operative Medicine, New Jersey Medical School, Rutgers, NJ USA; 3 Department of Anesthesiology, Zhongshan Hospital Affiliated to Xiamen University, Xiamen, China; 4 Department of Anesthesiology, Guangdong Province Hospital for Women and Children Health Care, Guangzhou, China

**Keywords:** Pharmacodynamics, Comparative study, Loss of consciousness, Sedation, Bispectral index monitor, Pharmakodynamik, Vergleichende Studie, Bewusstseinsverlust, Sedierung, Bispektrale Indexmonitor

## Abstract

**Background:**

The pharmacodynamics of propofol are closely linked to gender. Dexmedetomidine can decrease propofol needs during propofol anesthesia. The aim of this study was to compare the gender differences on the calculated effect site median effective concentration (EC_50_) of propofol for loss of consciousness (LOC) after pretreatment with different concentrations of dexmedetomidine.

**Methods:**

In this study 60 male and 60 female patients were randomly allocated to receive dexmedetomidine at target plasma concentrations of 0.0 ng/ml (0.0 group), 0.4 ng/ml (0.4 group), 0.6 ng/ml (0.6 group) and 0.8 ng/ml (0.8 group). Propofol was administered after dexmedetomidine had been intravenously infused for 15 min. The propofol infusion was targeted to provide an initial effect-site concentration of 1.0 μg/ml, followed by increments by 0.2 μg/ml when the effect-site concentration and target concentration of propofol were in equilibrium until LOC was established, where LOC was defined by the observer’s assessment of alertness/sedation scale (OAA/S) score < 2.

**Results:**

The calculated effect-site EC_50_ of propofol LOC was higher in males than in females in the 0.0, 0.4, 0.6, and 0.8 groups (2.43 vs. 2.17, 1.99 vs. 1.82, 1.72 vs. 1.56 and 1.50 vs. 1.32 μg/ml, respectively, all *p* < 0.05). The hypnotic interaction between dexmedetomidine and propofol could be described with an additive model of pharmacodynamic interaction.

**Conclusion:**

Gender significantly influenced the calculated effect-site EC_50_ of propofol for LOC after pretreatment with different concentrations of intravenous dexmedetomidine. It was concluded that an additive interaction could describe the results seen. Thus, gender has to be considered when these drugs are co-administered.

## Zusammenfassung

**Hintergrund:**

Die Pharmakodynamik von Propofol ist eng mit dem Patientengeschlecht verknüpft. Während einer Propofolanästhesie kann Dexmedetomidin den Propofolbedarf senken. Das Ziel dieser Studie war es, geschlechtsassoziierte Unterschiede der berechneten medianen effektiven Konzentration (EC_50_) von Propofol für einen Bewusstseinsverlust (LOC) nach Vorbehandlung mit verschiedenen Konzentrationen von Dexmedetomidin zu ermitteln

**Methoden:**

Je 60 männliche und weibliche Patienten wurden randomisiert einer von 4 Gruppen mit veschiedenen Dexmedetomidinzielkonzentrationen zugeordnet: 0,0 ng/ml (0,0 Gruppe), 0,4 ng/ml (0,4 Gruppe), 0,6 ng/ml (0,6 Gruppe) und 0,8 ng/ml (0,8 Gruppe). Nach einer 15-minutigen intravenösen Dexmedetomidininfusion wurde Propofol verabreicht. Die Propofolinfusion zielte auf eine initiale Konzentration am Wirkungsort von 1,0 μg/ml ab, gefolgt von Erhöhungen um 0,2 μg/ml, wenn sich die Konzentration am Wirkungsort und die Zielkonzentration von Propofol im Gleichgewicht befanden, bis ein LOC bei einem OAA/S(Observer’s Assessment of Alertness/Sedation Scale)-Score > 2 erreicht wurde.

**Ergebnisse:**

Die berechnete Propofol-EC_50_ am Wirkungsort für einen LOC war für Männern höher als für Frauen in den Gruppen 0,0, 0,4, 0,6 und 0,8 (2,43 vs. 2,17; 1,99 vs. 1,82; 1,72 vs. 1,56 und 1,50 vs. 1,32 μg/ml; alle *p* < 0,05). Die hypnotische Interaktion zwischen Dexmedetomidin und Propofol konnte mit einem additiven Modell einer pharmakodynamischen Interaktion beschrieben werden.

**Schlussfolgerungen:**

Das Geschlecht beeinflusst die berechnete EC_50_ von Propofol für einen LOC am Wirkungsort nach der Vorbehandlung mit verschiedenen Konzentrationen von i.v. Dexmedetomidin signifikant. Ein additives Interaktionsmodell konnte die beobachteten Ergebnisse beschreiben. Daher muss bei der gemeinsamen Verabreichung dieser Medikamente das Geschlecht berücksichtigt werden.

## Introduction

Propofol is a sedative and hypnotic agent commonly used for the induction and maintenance of general anesthesia because of its rapid onset and offset of effect. Dexmedetomidine, a useful and safe adjuvant in propofol anesthesia, is an α_2_-adrenoceptor agonist that provides sedation, anxiolysis, and analgesia. It has been shown that dexmedetomidine can blunt the cardiovascular response to tracheal intubation during propofol-based anesthesia [1, 2]. In addition, dexmedetomidine is frequently used to decrease propofol requirement and the incidence of post-operative delirium in propofol anesthesia [3, 4]. Hence, dexmedetomidine and propofol are often coadministered in general anesthesia.

Many studies have shown that the pharmacodynamics of drugs, for example propofol, are influenced by gender [5–7]. A recent study showed that male patients needed higher calculated effect-site median effective concentration (EC_50_, the concentration at which 50% of patients experience loss of consciousness, LOC) of propofol than female patients during supraglottic airway insertion in coadministration with 0.5 μg/kg body weight (BW) dexmedetomidine [8]. Based on these findings, gender should be considered during propofol anesthesia. Previous studies have shown that dexmedetomidine could reduce the calculated effect-site EC_50_ of propofol in a dose-dependent manner [9, 10]; however, it is not known if there is any gender disparity on the calculated effect-site EC_50_ of propofol during coadministration of different concentrations of dexmedetomidine. Therefore, the purpose of this study was to compare gender differences on the propofol-sparing effect of different concentrations of dexmedetomidine.

## Material and methods

### Subject selection

This study was approved by the Guangzhou General Hospital of PLA and was registered in ClinicalTrials.gov (registration number: NCT02853864). After obtaining written informed consent, 60 male and 60 female patients with an age range of 20–50 years old, an American Society of Anesthesiologists (ASA) score I–II and body mass index (BMI) of 18.0–25.0 kg/m^2^, were enrolled in the study. Excluded were patients with history of mental disorders, hearing impairment, severe systemic illness, substance abuse and bradyarrhythmia.

A randomization sequence was used to allocate both male and female patients to 4 dexmedetomidine concentration groups (0.0, 0.4, 0.6, and 0.8). Thus, there were a total of 8 groups each consisting of 15 subjects in this study.

### Protocol

A Philips MP30 monitor (Philips, Boeblingen, Germany) was used for measuring non-invasive mean arterial pressure (MAP), heart rate (HR), and the oxyhemoglobin saturation (SpO_2_) while a facemask delivered oxygen at a rate of 3 l/min. Bispectral index (BIS) values were monitored with Aspect Vista^TM^ (Model A-2000, BIS Aspect Medical Systems; Natick, MA, USA). The MAP was recorded every 3 min or after manual activation. The HR, SpO_2,_ and BIS values were monitored continuously. Patients were made non-peros (NPO) per ASA protocol. The forearm was used as the intravenous site to administer all drugs. Propofol was administered through target-controlled infusion (TCI) using a Fresenius infusion pump (Fresenius), with the PK-PD model of Schnider et al. [11]. Dexmedetomidine was given via TCI using a SN-50 infusion pump (Sino Medical-Device Technology) driven by STANPUMP software [12, 13].

**Fig. 1:**
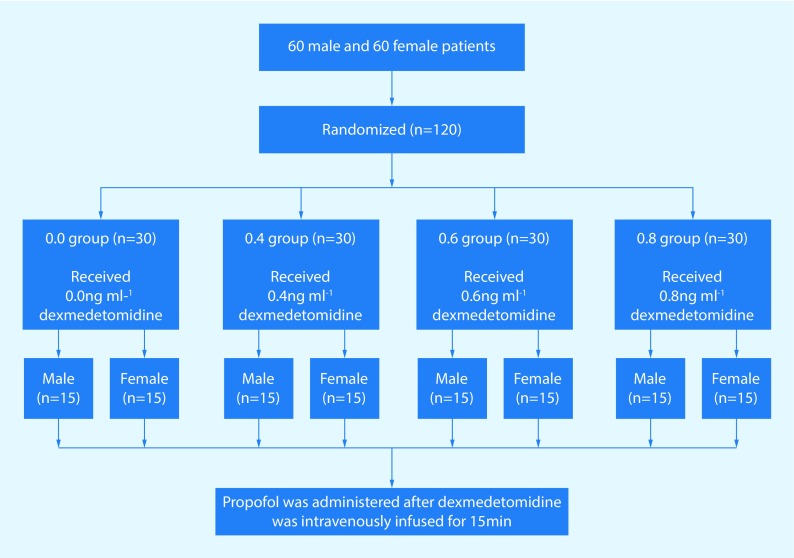
Disposition of the study patients

Patients were allocated to receive no dexmedetomidine in the 0.0 ng/ml group, a targeted effect-site concentration of dexmedetomidine 0.4 ng/ml in the 0.4 group, 0.6 ng/ml dexmedetomidine in the 0.6 group and 0.8 ng/ml dexmedetomidine in the 0.8 group by an independent observer blinded to the targeted dexmedetomidine concentration. Propofol was administered after dexmedetomidine had been infused intravenously for 15 min. (Fig. 1) The propofol administration was set to provide an initial effect-site concentration of 1.0 μg/ml, followed by increments of 0.2 μg/ml when the effect-site concentration and target concentration of propofol were balanced. After observing closure of patient’s eyes, the observer’s assessment of alertness/sedation scale (OAA/S) score was evaluated every 30 s [14] by an independent observer blinded to the dexmedetomidine concentration. An OAA/S score < 2 was regarded as LOC (absence of response to mild prodding or shaking) [15]. This study was terminated after LOC.

### Measurements

The calculated effect-site EC_50_ concentration of propofol and BIS were recorded following LOC.

### Statistical analysis

Statistical analysis was performed using SPSS 20.0. Results are expressed as mean and standard deviation (SD) if the data passed the normality test or, alternatively, as median and interquartile range. The one-way analysis of variance (normal data) or Jonckheere-Terpstra test for non-normal data was used to evaluate differences between groups. The ASA physical status was analyzed with the Fisher exact probability method. The EC_50_ and BIS_95_ (the BIS at which 95% of the patients achieved LOC) values were calculated using probit analysis. Comparison of EC_50_ between males and females in each dose group were analysed by use of the Mann-Whitney U‑test.

The mechanistic model of pharmacodynamic interaction was used to determine whether the interaction between dexmedetomidine and propofol was nonadditive or additive via unweighted least-squares nonlinear regression. The model [16] is described by equation A1:


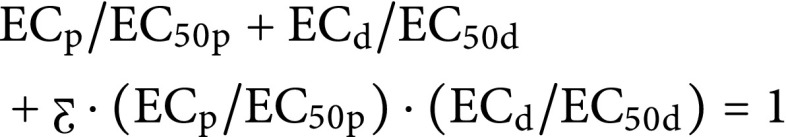


where EC_p_ is the effect-site concentration of propofol for LOC, EC_d_ is the plasma concentration of dexmedetomidine for LOC, EC_50p_ is the effect-site EC_50_ of propofol for LOC when it was given as a sole agent, EC_50d_ is the dexmedetomidine plasma concentration at which 50% of patients lost consciousness when it was administered as a sole drug and Ƹ is a dimensionless parameter characterizing the shape of the curve (with Ƹ ≠ 0 if the result is a curved line suggesting nonadditive interaction and Ƹ = 0 if the result is a straight line suggesting additivity).

The possibility of an additive interaction between dexmedetomidine and propofol was examined by the following equation [16] (derived from equation A1, assuming Ƹ = 0):

$$\mathrm{EC}_{\mathrm{p}}=\mathrm{EC}_{50\mathrm{p}}-\mathrm{EC}_{\mathrm{d}}\cdot \mathrm{EC}_{50\mathrm{p}}/\mathrm{EC}_{50\mathrm{d}}$$

The possibility of a nonadditive effect was examined by the following equation [16] (derived from equation A1, assuming Ƹ ≠ 0):


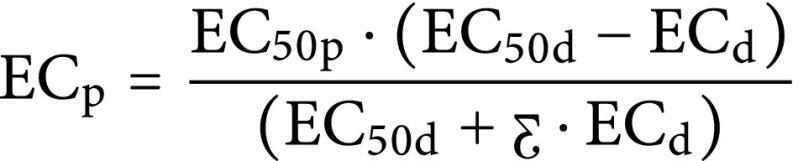


where EC_50p_, EC_50d,_ and Ƹ were calculated by nonlinear regression using the equation suggesting additivity and the equation suggesting a nonadditive effect. The residual sum of squares (RSS) of the curves was compared by an F‑test to determine which curve correlated best with the data used in the analysis. If the RSS of the fitted curve from the equation suggesting additivity was lower than that from the equation suggesting nonadditivity, the interaction between dexmedetomidine and propofol was judged to be additive. If no differences between the RSS of the two curves were found, the equations suggesting additivity and nonadditivity were similar and Ƹ was 0; therefore, the interaction between dexmedetomidine and propofol was judged to be additive. If judged to be nonadditive, the isobolographic method was used to analyze whether the interaction was synergistic or antagonistic. Comparison of EC_50p_ and EC_50d_ was analyzed by use of a t-test. *P* < 0.05 was regarded as a significance level for all tests which were two-tailed.

## Result

### Patient characteristics

All subjects completed the study. The characteristics of the patients are presented in Table 1. The eight groups were similar in terms of age, baseline BIS, ASA physical status and BMI (all *P* > 0.05).

**Table 1:** Demographics of the subjects participating in the study

Dose group		^a^Age (years)	^a^BMI (kg/m^2^)	ASA (I/II)	^a^Baseline BIS
0.0	Male	43.3 (6.2)	22.9 (1.5)	10/5	95.7 (1.9)
	Female	41.8 (6.2)	21.8 (2.1)	9/6	96.9 (1.6)
0.4	Male	41.1 (7.8)	22.3 (1.9)	9/6	95.8 (1.7)
	Female	44.3 (4.4.)	22.8 (1.9)	11/4	96.5 (2.0)
0.6	Male	41.7 (7.7)	22.3 (1.6)	10/5	96.1 (1.7)
	Female	43.5 (4.6)	22.6 (2.0)	11/4	96.1 (2.1)
0.8	Male	40.1 (7.3)	23.1 (1.7)	9/6	95.7 (1.6)
	Female	42.5 (6.7)	21.8 (1.6)	11/4	95.0 (1.7)

*BMI* body mass index, *ASA* American Society of Anesthesiologists grade, *BIS* bispectral index

^a^Expressed as mean and SD

#### Hemodynamics

**Fig. 2:**
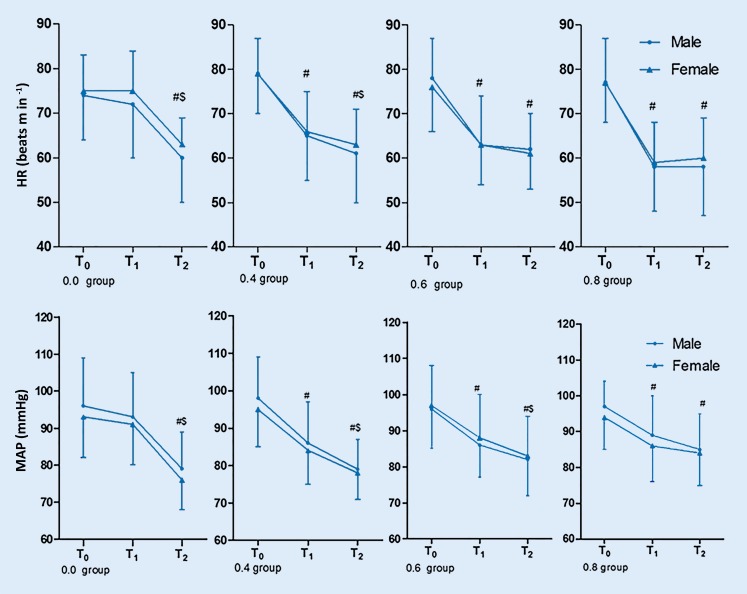
Graphs showing comparisons of the heat rate (HR) and mean arterial pressure (MAP) between males and females in the four groups in the study. *T0* is the time point at baseline, *T1* is the time point of starting the administration of dexmedetomidine for 15 min, and *T2* is the time point of LOC. *#* *P* < 0.05 compared with T0, *$* *P* < 0.05, comparison of HR and MAP between T1 and T2 in males and females

In groups 0.0 and 0.4, following induction of LOC by propofol, HR and MAP continued to decline. The MAP at LOC significantly declined in the 0.6 group but HR remained stable. In the 0.8 group both HR and MAP remained stable at LOC. There were no significant differences between genders in any of the dose groups (*P* > 0.05) (Fig. 2).

### Propofol EC_50_ for LOC

The calculated effect-site EC_50_ of propofol for LOC in male patients was 2.43μg/ml (range 2.36–2.49μg/ml) in the 0.0 group, 1.99μg/ml (1.95–2.03μg/ml) in the 0.4 group, 1.72μg/ml (1.68–1.76μg/ml) in the 0.6 group and 1.50μg/ml (1.45–1.54μg/ml) in the 0.8 group. The calculated effect-site EC_50_ of propofol for LOC in female patients was 2.17μg/ml (2.11–2.23μg/ml) in the 0.0 group, 1.82μg/ml (1.78–1.86μg/ml) in the 0.4 group, 1.56μg/ml (1.52–1.59μg/ml) in the 0.6 group, and 1.32μg/ml (1.27–1.36μg/ml) in the 0.8 group. Female patients had a lower calculated effect-site EC_50_ of propofol for LOC compared with male patients in the 0.0 group, 0.4 group, 0.6 group, and 0.8 group (all, *P* < 0.05) (Table 2).

**Table 2:** Propofol effect-site EC_50_ and EC_95_ for LOC according to group and gender

Dose group	EC_50_/EC_95_ (95%CI) for males (μg/ml)	EC_50_/EC_95_ (95% CI) for females (μg/ml)
0.0	2.43 (2.36–2.49)/3.01 (2.87–3.26)	2.17 (2.11–2.23)/2.64 (2.50–2.90)^d^
0.4	1.99 (1.95–2.03)/2.33 (2.25–2.47)^a^	1.82 (1.78–1.86)/2.21 (2.11–2.38)^a^
0.6	1.72 (1.68–1.76)/2.05 (1.97–2.18)^a, b^	1.56 (1.52–1.59)/1.86 (1.78–2.00)^a, b, c^
0.8	1.50 (1.45–1.54)/1.90 (1.80–2.07)^a, b, c^	1.32 (1.27–1.36)/1.70 (1.60–1.91)^a, b, c, d^

*LOC* loss of consciousness, *EC*_*50*_ effective concentration of propofol at which 50% of patients experience loss of consciousness, *EC*_*95*_ effective concentration of propofol at which 95% of patients experience loss of consciousness, *CI* confidence interval

^a^Significant difference compared with 0.0 group (*P* < 0.05)

^b^Significant difference compared with 0.4 group (*P* < 0.05)

^c^Significant difference compared with 0.6 group (*P* < 0.05)

^d^Significant difference between male and female patients (*P* < 0.05)

### Analysis of the interaction for LOC

No significant differences were found between the RSS of the models exploring the possibility of a nonadditive or additive interaction between dexmedetomidine and propofol for male patients (3,000,285 vs. 2,999,340, *P* > 0.05). The interaction for LOC between dexmedetomidine and propofol in males was judged to be additive. The RSS of the model exploring the possibility of an additive interaction between dexmedetomidine and propofol for female patients was similar to that of the model exploring the possibility of a nonadditive interaction (2,969,093 vs. 2,962,124,* P* > 0.05). The interaction for LOC between dexmedetomidine and propofol in females was judged to be additive. The calculated effect-site EC_50_ of propofol for LOC was higher in males than in females (2.43 vs. 2.17 μg/ml, *P* < 0.05). The calculated plasma EC_50_ of dexmedetomidine for LOC in males and females was similar (2.06 vs. 2.09 ng/ml,* P* > 0.05) (Table 3).

**Table 3:** Estimated values of the concentrations of propofol and dexmedetomidine associated with EC_50_ for LOC

Interaction	EC_50p_ (95% CI) (μg/ml)	EC_50d_ (95% CI) (ng/ml)	Ƹ (95% CI)	R^2^	RSS
Additivity^a^	2.50 (2.39–2.60)	2.06 (1.78–2.33)	0	0.71	3,000,285
Nonadditivity^a^	2.50 (2.38–2.62)	2.16 (−0.04–4.36)	0.08 (−1.58–1.74)	0.71	2,999,340
Additivity^b^	2.26 (2.16–2.37)*	2.09 (1.79–2.40)	0	0.67	2,969,093
Nonadditivity^b^	2.25 (2.13–2.37)	1.62 (0.58–2.67)	−0.35 (−1.13–0.43)	0.68	2,962,124

*R*^*2*^ the correlation coefficients for two possible interactions for LOC, *Ƹ* a dimensionless parameter characterizing the shape of the curve, *RSS* the residual sum of squares, *EC*_*50p*_ effective concentration of propofol at which 50% of patients experience loss of consciousness, *EC*_*50d*_ effective plasma concentration of dexmedetomidine at which 50% of patients lost consciousness when administered as a sole drug

*P < 0.05 for comparison of EC_50_ between males and females

^a^ Exploring the possibility of an additive or nonadditive interaction for male patients

^b^ Exploring the possibility of an additive or nonadditive interaction for female patients

### BIS_95_ for LOC

The Bis_95_ is a statistical term defining that 95% will have LOC at this BIS value. In this study the Bis_95_ for male patients was equal to 55 (95% CI: 53–56) in the 0.0 group, 57 (95% CI:55–59) in the 0.4 group, 57 (95% CI: 54–58) in the 0.6 group and 58 (95% CI: 55–60) in the 0.8 group. The BIS_95_ for female patients was 56 (95% CI: 54–57) in the 0.0 group, 58 (95% CI:54–59) in the 0.4 group, 59 (95% CI: 58–61) in the 0.6 group and 59 (95% CI: 56–61) in the 0.8 group. No significant differences between gender groups were found (*P* > 0.05) (Table 4).

**Table 4:** The mean BIS values (confidence interval) at which patients had a 95% probability of LOC

	0.0 group	0.4 group	0.6 group	0.8 group
Male	55 (53–56)	57 (55–59)	57 (54–58)	58 (55–60)
Female	56 (54–57)	58 (54–59)	59 (58–61)	59 (56–61)

*BIS* bispectral index, *LOC* loss of consciousness

## Discussion

Propofol is a common hypnotic-sedative drug with rapid onset and offset of effect; however, when used alone it causes dose dependent cardiorespiratory depression [17]. Various adjunct medications including opioids, benzodiazepines and α_2_ agonists have been employed as co-induction agents to change the adverse effects of propofol. [18, 19]. Dexmedetomidine is frequently used to reduce propofol requirement during propofol anesthesia. This study compared gender difference on the propofol-sparing effect of dexmedetomidine after pretreatment with different concentrations. The primary outcome parameter studied was the calculated effect-site median effective concentration (EC_50_) of propofol after pretreatment with different concentrations of dexmedetomidine. The EC_50_ of propofol is defined as the effect site concentration at which 50% of patients experience loss of consciousness (LOC). It was found that there were no significant differences in the plasma EC_50_ of dexmedetomidine between males and females (2.06 vs. 2.09 ng/ml; *p* > 0.05). There were, however, significant gender differences between the calculated effect-site EC_50_ of propofol. Males required higher calculated effect-site EC_50_ than females in all 4 groups of 0.0, 0.4, 0.6 and 0.8 ng/ml. This is consistent with Kodaka et al. who found similar results that males patients needed significantly higher propofol concentrations to achieve LOC than females (2.9 vs. 2.7 μg/ml *p* < 0.05) [6]. Another study also reported that male patients required higher calculated effect-site EC_50_ of propofol during i‑gel (LMA) insertion after pretreatment with 0.5 μg/kg of dexmedetomidine (5.46 vs. 3.82 μg/ml, *P* < 0.01) [8]. In contrast to the study by Choi et al. different concentrations of dexmedetomidine were preadministered, namely 0.0, 0.4, 0.6, and 0.8 ng/ml over 15 mins before starting a TCI infusion of propofol to achieve an initial effect-site concentration of 1.0 μg/ml. This was followed by increments of 0.2 μg/ml until the clinical end point of LOC was confirmed. Rapid administration of the induction dose of propofol is associated with a significant reduction in blood pressure of approximately 20–40%. Thus, the effect site EC_50_ of propofol was gradually increased [20].

According to the mechanistic model, the pharmacodynamic interactions of dexmedetomidine and propofol for induction of LOC were additive regardless of gender. No significant differences were found between genders for plasma EC_50d_ of dexmedetomidine. (Table 3) Dexmedetomidine induces hyperpolarization of noradrenergic locus ceruleus neurons to enhance eye movement sleep promoting pathways to achieve sedation. [21]. As found in a recent study by Zhao et al. [10], this study showed that dexmedetomidine significantly and dose-dependently decreased the calculated effect-site EC_50_ of propofol and BIS values during anesthesia induction. The findings contrast with a previous study which concluded that the calculated effect-site EC_50_ of propofol required to produce adequate anesthesia for esophagoduodenoscopy (EGD) in children was unaffected by a concomitant infusion of dexmedetomidine 1 μg/kg given over 10 min [22]. It is possible that the concomitant administration of propofol and dexmedetomidine in the study did not allow adequate time for dexmedetomidine to exert a propofol-sparing effect. It may also well be that dexmedetomidine lacks a propofol-sparing effect in the pediatric population. Further research will be elucidative.

The sedative effect of dexmedetomidine has been shown to be more significant in female patients during the luteal phase of menstrual cycles than the follicular phase [23]. Progesterone and its metabolites are known to have sedative, anxiolytic, analgesic and anticonvulsant effects [24–26]. A recent study demonstrated that the progesterone levels were inversely correlated with the calculated effect site EC_50_ of propofol [27]. Propofol and progesterone share similar mechanisms of action through the gamma-aminobutyric acid type A (GABA_A_) receptor, which is known to be among the major binding sites for several general anesthetics [28, 29]. Hence the propofol-sparing effect during the luteal phase is most likely an additive effect given that the luteal phase has progesterone dominance and so dexmedetomidine will exert a more sparing effect on propofol. In contrast, another study found that intraoperative administration of dexmedetomidine had a stronger morphine-sparing effect in postoperative pain control in males compared to females [30]. A possible mechanism for this finding is unknown although animal studies implicated estrogen which is believed to attenuate α_2_ adrenergic receptor-mediated analgesia [31]. This study, just like the present study, did not account for the menstrual cycle phases of the female participants.

The bispectral index was introduced by Aspect Medical System in 1994 to facilitate objective evaluation of depth of sedation [32]. It assesses the level of consciousness by algorithm analysis of patient’s electroencephalographic data during general anesthesia [33]. This study did not find an effect of gender on the BIS_95_ values for LOC as defined (Table 4). This is consistent with a previous study which showed that men and women had equivalent BIS values at LOC_50_ [6]. Therefore, the BIS can be used to monitor depth of anesthesia and anesthetic dosing and to avoid an unnecessary deep anesthetic state as well as an increased risk of awareness during combined propofol/dexmedetomidine anesthesia.

This study has certain limitations. First, higher concentrations of dexmedetomidine were not used as these concentrations are known to increase pulmonary arterial pressure and vascular resistance which will lead to bradycardia and hypotension [34–36]. Thus, a maximum of 0.8 ng/ml plasma concentration of dexmedetomidine was used to avoid serious cardiovascular effects. Second, plasma drug concentrations of propofol and dexmedetomidine were not measured. Predicted concentrations were used for data analysis. Last, the study did not account for the menstrual cycle phase of the female study participants.

In conclusion, the present study shows that gender has association with the calculated effect-site EC_50_ of propofol for LOC (defined as OAA/S score < 2) after pretreatment with different concentrations of dexmedetomidine. According to this, gender should be considered when these drugs are co-administered during anesthesia induction.
